# RNA and epigenetic silencing: Insight from fission yeast

**DOI:** 10.1111/j.1440-169X.2011.01310.x

**Published:** 2012-01

**Authors:** Derek B Goto, Jun-ichi Nakayama

**Affiliations:** 1 Creative Research Institution, Hokkaido UniversitySapporo 001-0021; 2 Laboratory for Chromatin Dynamics, RIKEN Center for Developmental BiologyKobe, Hyogo 650-0047, Japan

**Keywords:** epigenetic silencing, fission yeast, heterochromatin, histone methyltransferase, RNA interference

## Abstract

Post-translational modifications of histones are critical not only for local regulation of gene expression, but also for higher-order structure of the chromosome and genome organization in general. These modifications enable a preset state to be maintained over subsequent generations and thus provide an epigenetic level of regulation. Heterochromatic regions of the genome are epigenetically regulated to maintain a “silent state” and protein coding genes inserted into these regions are subject to the same epigenetic silencing. The fission yeast *Schizosaccharomyces pombe* has well characterized regions of heterochromatin and has proven to be a powerful model for elucidation of epigenetic silencing mechanisms. Research in *S. pombe* led to the breakthrough discovery that epigenetic silencing is not solely a chromatin-driven transcriptional repression and that RNA interference of nascent transcripts can guide epigenetic silencing and associated histone modifications. Over the last 10 years, an eloquent integration of genetic and biochemical studies have greatly propelled our understanding of major players and effector complexes for regulation of RNAi-mediated epigenetic silencing in *S. pombe*. Here, we review recent research related to regulation of the epigenetic state in *S. pombe* heterochromatin, focusing specifically on the mechanisms by which transcription and RNA processing interact with the chromatin modification machinery to maintain the epigenetically silent state.

## Introduction

The organization of chromosomal DNA within the nucleus is achieved through direct interaction with a wide range of proteins. The basic unit of the DNA-protein complex, known as chromatin, is the nucleosome that contains approximately 147 bp of DNA wrapped around a core of eight histone proteins: two copies each of the H2A, H2B, H3 and H4 histone proteins. Post-translational modification of core histone proteins and further interactions with nuclear proteins regulates the nature of chromatin and its subsequent activity. In general, the majority of active protein-coding genes are located within euchromatin, regions of chromatin in which the nucleosome particles have an “open” packaging structure amenable to access by regulatory proteins and the transcriptional machinery. In contrast, inactive genes and structural regions of the genome, such as the centromeres and telomeres, are packaged with the nucleosome in a more tight “closed” state known as heterochromatin. The post-translational modifications of chromatin that define its state enable a preset gene activity to be maintained even in the absence of a regulatory signal. This is also a heritable state that can be transferred to subsequent generations without requiring change of DNA sequence, and is thus referred to as epigenetic regulation.

**Fig. 1:**
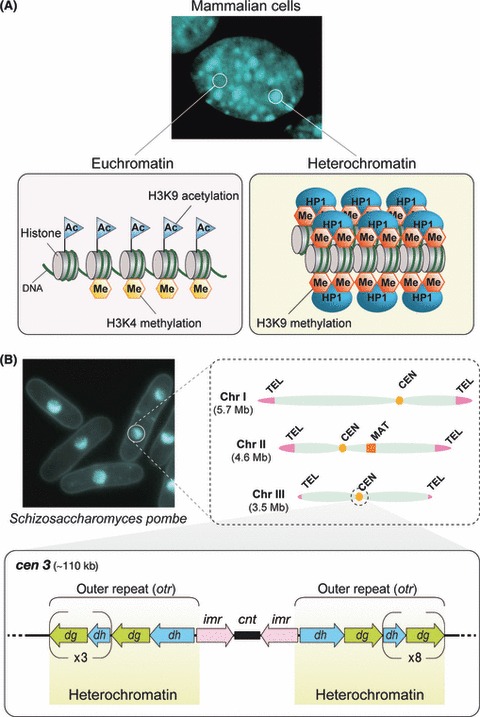
Heterochromatin and epigenetically silent regions of the genome. (A) Active protein coding genes are generally contained within euchromatin, typically modified by acetylation (Ac) of histone H3 lysine 9 (H3K9) and methylation (Me) of histone H3 lysine 4 (H3K4). In contrast, heterochromatin is generally considered to have a compact structure and can be observed as densely stained regions in nuclei. Heterochromatin is characterized by H3K9 methylation, which is recognized by Heterochromatin Protein 1 (HP1). The upper panel shows one complete 4′6′-diamidino-2-phenylindole dihydrochloride (DAPI)-stained nucleus (center) of a mouse NIH3T3 cell. (B) *Schizosaccharomyces pombe* chromosomes and heterochromatin. The *S. pombe* genome is organized across three chromosomes, with heterochromatin found at the telomeres (TEL), centromeres (CEN) and mating type region (MAT). Centromeric regions (lower panel) are arranged with a unique core centromere region (*cnt* and *imr*) flanked by heterochromatin covering the pericentromeric outer repeat regions (*otr*). The *otr* contains multiple copies of *dg* and *dh* repeat sequences that vary in number depending on the chromosome. The upper left panel shows *S. pombe* cells stained with Hoechst33342.

The N-terminal regions of histone proteins, commonly called the “histone tails” as they extend from the nucleosomes, are highly conserved among eukaryotic organisms and represent the target site for most common epigenetic modifications. The major post-translation modifications involved in epigenetic regulation are found in the histone H3 tails, particularly at the lysine 4 (H3K4) and lysine 9 (H3K9) residues. The chromatin at actively transcribed genes and associated euchromatic regions is marked by methylation of lysine 4 (H3K4me) and acetylation of lysine 9 (H3K9ac). The opposite is found in heterochromatin, where methylation of lysine 9 (H3K9me) and subsequent coating by Heterochromatin Protein 1 (HP1) proteins is a hallmark of epigenetically silent chromatin (Fig. 1). It is important to note that these are not the only modifications found in histone tails. A large number of modifications can exist depending on the specific context or DNA process, and positive and negative interactions between different modifications also occur (for review see Kouzarides 2007). Phosphorylation of histone H3 serine 10 (H3S10) contributes to disruption of heterochromatin structure during the cell cycle (Dormann *et al.* 2006), whereas methylation of H3K27 by the Polycomb machinery is associated with epigenetic suppression of developmental genes in multicellular organisms (Schuettengruber & Cavalli 2009). In the case of plants and vertebrates, methylation of DNA cytosine residues also plays a significant role in epigenetic control of gene expression.

Although heterochromatic regions of the genome contain few protein-coding genes, the mechanisms for epigenetic silencing are directly relevant to that occurring at protein-coding genes and for the control of mobile transposon elements that can strongly influence neighboring genes. Epigenetically silent heterochromatin also serves an essential function in chromosome dynamics, supporting kinetochore formation to ensure correct chromosome segregation and for maintenance of telomere integrity at chromosome ends. Mutations that disrupt any aspect of epigenetic silencing, such as those that impair H3K9me or reduce HP1 association with chromatin, often display phenotypes of lagging chromosomes and missegregation, and in severe cases cell lethality (Ekwall *et al.* 1995; Peters *et al.* 2001).

The fission yeast *Schizosaccharomyces pombe* serves as a highly effective model for the study of basic cellular processes. Research in *S. pombe* has provided significant insight on the regulation of the cell cycle, and it was discoveries in this area that led to Sir Paul Nurse being awarded the Nobel Prize in Physiology or Medicine 2001. *S. pombe* also shares many of the chromatin modifications with higher organisms. Chromatin research in *S. pombe* benefits from the fact that the structural organization of the centromeres and associated histone modifications are more complex than that in budding yeast and similar to that of multicellular organisms, yet it contains only three chromosomes and in many cases only one copy of key regulatory proteins. The centromeres in *S. pombe* are arranged with a core centromere region containing a central sequence (*cnt*) flanked by two inverted inner repeats (*imr*) that share exact identity, which is further flanked by the pericentromeric outer repeat region (*otr)* that contains multiple copies of *dg* and *dh* repeat sequences (Fig. 1). The core region (*cnt* and *imr*) is unique to each chromosome whereas the pericentromeric repeats of each chromosome *otr* are similar and vary in number depending on the chromosome (Takahashi *et al.* 1992; Rhind *et al.* 2011). The *cnt* acts to form the kinetochore for spindle attachment during mitosis and has a chromatin structure specific to this purpose (for review see Allshire & Karpen 2008). On the other hand, the pericentromeric repeats function as sites of typical heterochromatin formation and are essential for higher order structure and correct function of the centromere itself.

Due to the above characteristics, *S. pombe* is a powerful tool to elucidate epigenetic silencing mechanisms of heterochromatin (Martienssen *et al.* 2005) and to infer the relationship of heterochromatin formation with centromere function (Pidoux & Allshire 2004). In 2002, research with *S. pombe* provided the breakthrough discovery that RNAi-mediated processing of native RNA transcripts can guide epigenetic silencing and histone modification (Volpe *et al.* 2002). This prompted a new field of research and parallels have now been found in multicellular organisms including plants, flies, and vertebrate cells (Huisinga & Elgin 2009; Matzke *et al.* 2009; Bourc’his & Voinnet 2010; Malecova & Morris 2010). Although the requirement for transcription and RNAi in heterochromatic silencing was immediately apparent, the molecular mechanisms behind this regulation and the nature of interactions between different protein components remained elusive. An eloquent combination of genetic and biochemical studies by different laboratories is now enabling a clearer picture of how this epigenetic silencing is formed at heterochromatic regions and inherited through mitotic division.

## The breakthrough: a role for RNA in epigenetic silencing

In addition to the centromeric and pericentromeric regions, the *S. pombe* genome contains the mating type (*mat*) region that is another well studied region of heterochromatin. The mating type region contains an upstream active *mat1* locus and two downstream donor loci, *mat2* and *mat3*, within a silent heterochromatin domain. Switching of the upstream *mat1* gene with one of two downstream donor loci determines the mating type of the cell. Correct regulation of heterochromatin silencing is essential for efficient mating type switching. This region long served as a target site for heterochromatin studies in *S. pombe* (Klar 2007), and it was the results from early genetic screens using this locus that enabled identification of what are still considered to be the major key players for chromatin modification in *S. pombe*, particularly *swi6*, *rik1*, and *clr4* (Ekwall *et al.* 1995, 1996). Early analyses of mutants resulting from these genetic screens all pointed to a mechanism for epigenetic silencing based on transcriptional gene silencing (TGS). However, it is now clear that redundant mechanisms operate at the mating type locus (Jia *et al.* 2004). This redundancy had been continually masking the presence of another unexpected mechanism for heterochromatin silencing that is readily observable at the pericentromeric repeats, one that requires nascent RNA transcripts and involves a post-transcriptional gene silencing (PTGS) activity.

RNA interference (RNAi) is a PTGS regulation that either represses translation of target transcripts or results in their cleavage and rapid degradation. The RNAi process is mediated by small RNAs, microRNAs or short interfering RNAs (siRNAs), that have complementary sequence to the target transcript and determine the site of action for the RNA machinery (Hannon 2002; Mello & Conte 2004; Ghildiyal & Zamore 2009). Small RNAs are generated from longer double stranded RNAs by the ribonuclease III enzyme Dicer and then loaded into the Argonaute protein, which functions in a silencing complex and binds target transcripts using the small RNA as a guide (Hammond *et al.* 2000; Song *et al.* 2003). PTGS that involves cleavage of the target is achieved by an endonuclease “slicing” activity contained within the Argonaute protein itself (Song *et al.* 2004; Irvine *et al.* 2006). An additional activity associated with RNAi is that of RNA-dependent RNA polymerase (RdRP). Found in plants, fungi and invertebrate animals, this is considered an amplification or enhancement step where RdRP synthesizes a complementary strand of the target transcript to form dsRNA that serves as a template for Dicer and additional small RNA production (Cogoni & Macino 1999; Dalmay *et al.* 2000; Smardon *et al.* 2000; Sijen *et al.* 2001). Prior to 2002, a role for PTGS in the nucleus was unknown and RNAi was thought of as a cytoplasmic regulation of protein-coding transcripts that was unrelated to chromatin state.

The discovery that RNAi may participate in epigenetic silencing was the result of clever foresight originating from research into control of plant development. *Argonaute* genes were suggested to have an important role in cell differentiation in plants (Kidner & Martienssen 2004), and to gain further insight on how these proteins function, this research was extended into *S. pombe* due to the fact that its genome only contained a single copy of *Argonaute* (*ago1*^*+*^). Deletion of *ago1*^*+*^ and the related single *Dicer* (*dcr1*^*+*^) and *RdRP* (*rdp1*^*+*^) genes led to the surprising finding that these core components of the RNAi machinery were required for maintaining the silent state of pericentromeric regions (Volpe *et al.* 2002). Further analysis revealed that, contrary to common belief, one strand of the heterochromatin DNA was actually being transcribed in wild-type cells and this was being turned-over by the RNAi machinery. RNAi processing of this strand was required to maintain TGS of the opposite strand that was enforced by typical chromatin modifications. Loss of the RNAi machinery resulted in transcription from both strands of the pericentromeric repeats and a reduction of H3K9me at transgenes inserted within heterochromatin, demonstrating the requirement for RNAi in maintaining the epigenetic state (Volpe *et al.* 2002). This seminal discovery revealed that the two seemingly unrelated fields of RNA processing and chromatin modification could be tightly integrated in key developmental processes, and was accordingly recognized as the “Breakthrough of the Year” for 2002 (Couzin 2002).

While the above discovery demonstrated a role for RNAi in epigenetic silencing, the nature of this role and mechanisms for interaction between RNAi and chromatin machineries were less clear. Intensive biochemical and genetic experiments by several groups over the following years have revealed that many of the core RNAi and chromatin regulatory proteins function in concert with each other as larger complexes. Each of these complexes has a specific function in RNAi-mediated epigenetic silencing as described in the section below.

### Silencing by not being silent

Due to its highly compact structure, heterochromatin was long considered to be inaccessible to the transcriptional machinery and thus completely devoid of transcription activity. It is now known that this is not necessarily the case, and that nascent RNA polymerase II-dependent transcripts from low levels of strand-specific transcription are required to maintain the epigenetically silent state (Volpe *et al.* 2002; Djupedal *et al.* 2005; Kato *et al.* 2005). Although this appears to be a paradox at first glance, the contradiction arises by a definition of silencing as simply whether transcription takes place or not. This can easily be resolved by a clearer definition of epigenetic silencing. Indeed, although low levels of strand-specific transcription may occur across transgenes located within heterochromatic regions (Volpe *et al.* 2002; Irvine *et al.* 2006), these protein-coding genes are not transcribed in a way that facilitates translation and thus remain functionally silent (Allshire *et al.* 1995). Epigenetic silencing may therefore better be defined as an epigenetic regulation that maintains a functionally silent state, irrespective of whether it is achieved through a transcription-independent (TGS) or transcription-dependent (PTGS) activity, or a combination of both.

## Current major complexes and their activities

The list of proteins known to be involved in RNAi-mediated epigenetic silencing has increased considerably in the last few years, and biochemical characterization is providing an insight into their functions. At present, four major complexes have been identified that are required for maintaining epigenetic silencing at heterochromatin. When considering the biological functions of these complexes, it is important to keep in context the regions of heterochromatin studied. Epigenetic silencing may be maintained differently within a heterochromatin region depending on the nature of specific sequence being examined. When a transgene is inserted within the pericentromeric region, heterochromatin spreads over the gene and it becomes epigenetically silenced similar to that for the repeats. The presence of H3K9me and epigenetic silencing at inserted transgenes is almost entirely dependent on the RNAi machinery, consistent with RNAi having an upstream role and recruiting the chromatin modification machinery to these sequences (Volpe *et al.* 2002; Sadaie *et al.* 2004; Verdel *et al.* 2004; Irvine *et al.* 2006). However, at endogenous pericentromeric repeat sequences, H3K9me and heterochromatin can still be maintained at specific sites by the action of the H3K9 methyltransferase Clr4 even when silencing of both strands is released by mutation of RNAi (Noma *et al.* 2004; Sadaie *et al.* 2004; Li *et al.* 2008). In this context, RNAi appears to have a role to enforce silencing and spread heterochromatin to neighboring regions after initial recruitment by chromatin modification machinery (Kagansky *et al.* 2009). Both observations were supported by an analysis of Ago1 slicing activity, which demonstrated that catalytic inactive Ago1 could still associate with repeat sequences, whereas Ago1-dependent processing of read-through transcripts from the repeats was required for its localization to inserted transgenes and to maintain H3K9me at these sequences (Irvine *et al.* 2006).

### The RNAi-induced transcriptional silencing complex

**Fig. 2:**
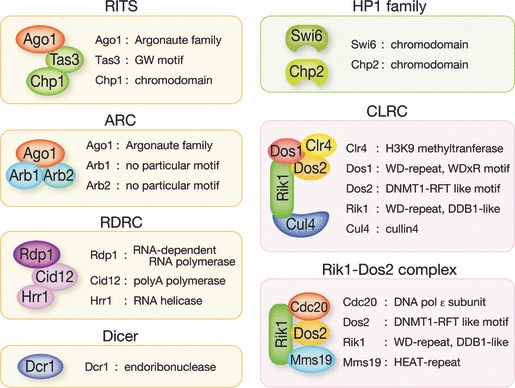
Proteins and effector complexes linked to RNAi-mediated epigenetic silencing in *Schizosaccharomyces pombe*. Complexes containing known RNAi components are shaded in yellow, whereas complexes interacting directly with chromatin are shaded in pink. Heterochromatin Protein 1 (HP1) proteins are also a key component of heterochromatin structure and are included for consistency.

In *S. pombe*, four of the nine chromodomain proteins contained within its genome (Wood *et al*. 2002) have been confirmed to have a functional role in heterochromatin modification: Clr4 H3K9 methyltransferase, HP1 proteins Chp2 and Swi6, and a unique chromodomain protein Chp1 (Ekwall *et al.* 1995; Doe *et al.* 1998; Ivanova *et al.* 1998; Partridge *et al.* 2000, 2002; Thon & Verhein-Hansen 2000; Bannister *et al.* 2001; Nakayama *et al.* 2001; Sadaie *et al.* 2004, 2008; Motamedi *et al.* 2008). A critical link in understanding how RNAi activity interacts with heterochromatin was provided by a biochemical analysis of tagged Chp1 purified from *S. pombe* cells. This revealed that Chp1 directly interacts with the RNAi Ago1 protein and forms a complex that includes a third protein named Tas3 (Verdel *et al.* 2004). The Chp1-Tas3-Ago1 complex interacts with siRNAs derived from pericentromeric sequences in a Dcr1-dependent manner, and is required for epigenetic silencing and H3K9me at transgenes within the pericentromeric region. Based on these features, this complex was named as RNAi-induced transcriptional silencing (RITS) complex (Fig. 2) (Verdel *et al.* 2004). The association of RITS with heterochromatin depends on H3K9me through the Chp1 subunit, although in the absence of *dcr1*^*+*^, siRNA loading or Ago1 catalytic activity, the inactive RITS does not spread H3K9me into transgenes or repress accumulation of heterochromatin transcripts and epigenetic silencing is released (Noma *et al.* 2004; Verdel *et al.* 2004; Irvine *et al.* 2006). RITS therefore appears to function to recruit the RNAi machinery to heterochromatin for reinforcement of the silencing and guide the spread of epigenetic silencing across the entire region.

### The RNA-dependent RNA polymerase complex

The RNAi process depends on dsRNA that is cleaved by Dcr1 to provide siRNAs for loading into Ago1. Although one strand of the pericentromeric repeats is weakly transcribed, TGS of the opposite strand in wild-type cells means an absence of a direct complementary strand for these transcripts. It could be argued that the repetitive nature of pericentromeres may inadvertently generate an appropriate source of dsRNA; however, this would not explain how RITS and slicing activity of Ago1 is able to expand into non-repetitive transgene sequences. The *S. pombe* genome encodes an RNA-dependent RNA polymerase, Rdp1, that functions in a complex with a RNA helicase, Hrr1, and a polyA polymerase family protein, Cid12, to synthesize dsRNA complementary strands from target transcripts (Fig. 2) (Motamedi *et al.* 2004). The RNA-dependent RNA polymerase complex (RDRC) is not tightly associated with chromatin like RITS; however, an association between the two complexes can be detected and loss of siRNAs from RITS in RDRC mutants is consistent with this complex functioning to provide the Dcr1-dependent siRNA substrate for RITS (Motamedi *et al.* 2004; Iida *et al.* 2008). As it is being synthesized by RDRC, it is likely that the dsRNA substrate is provided directly to Dcr1 for siRNA production in a coupled process. Dcr1 can associate with RDRC through a domain independent from its RNaseIII activity and this interaction does not require dsRNAs or heterochromatin assembly (Colmenares *et al.* 2007). As suggested by the presence of Cid12 within RDRC, select components of the spliceosome also functionally contribute to RDRC function and epigenetic silencing at the pericentromeric region, although this is not coupled to splicing activity and likely represents a structural support for RNA processing by the RNAi machinery (Bayne *et al.* 2008; Chinen *et al.* 2010).

### The Argonaute siRNA chaperone complex

Epigenetic silencing by the RITS complex at heterochromatin requires that it is loaded with relevant siRNAs. The Argonaute siRNA chaperone (ARC) complex is a second Ago1-containing complex that is proposed to mediate the loading of Ago1 with mature single-stranded siRNA for its activity within the RITS complex (Fig. 2) (Buker *et al.* 2007). The ARC complex contains two other conserved proteins, Arb1 and Arb2, and differs from RITS in that it is not localized to chromatin and shows diffuse distribution in both the cytoplasm and nucleus. Duplex siRNAs are predominantly found in the ARC complex and the maturation and conversion of these to single-stranded siRNAs requires the slicer activity of Ago1. The Arb1 subunit shows suppression activity against Ago1 slicing catalytic activity, suggesting that the ARC complex functions to obtain duplex siRNAs from Dcr1 and prevent their autodegradation until they are required as single-stranded siRNAs in the RITS complex (Buker *et al.* 2007).

### The CLR4 complex

The accumulation of high levels of siRNA derived from the heterochromatin repeats requires functional Clr4, the sole H3K9 methyltransferase in *S. pombe* (Motamedi *et al.* 2004; Noma *et al.* 2004; Hong *et al.* 2005; Buhler *et al.* 2006; Halic & Moazed 2010). Clr4 is present in a complex containing the Rik1 protein, Rik1-associated factors Dos1/Raf1 and Dos2/Raf2, and the ubiquitin ligase scaffold family protein cullin 4 (Cul4) (Fig. 2; Sadaie *et al.* 2004; Horn *et al.* 2005; Li *et al.* 2005). Deletion of the CLR4 complex (CLRC) subunits releases epigenetic silencing of both strands of pericentromeric repeats and abolishes H3K9me, confirming its upstream role in heterochromatin formation and epigenetic silencing (Sadaie *et al.* 2004; Hong *et al.* 2005; Horn *et al.* 2005; Jia *et al.* 2005; Li *et al.* 2005; Thon *et al.* 2005). Consistent with this, siRNAs derived from the pericentromeric repeats fail to accumulate in a CLRC mutant background (Hong *et al.* 2005; Li *et al.* 2005).

Although Clr4 was identified as the namesake component of CLRC, stoichiometrical validation will be necessary to understand exactly how the complex functions to support Clr4 activity and H3K9me. In several purification studies, Rik1, Cul4, and other CLRC components were stably identified by mass spectrometric analyses, but Clr4 was missing (Horn *et al.* 2005; Bayne *et al.* 2010). Based on amino-acid similarity, Rik1 is thought to have an intertwined three β-propeller cluster and to be a functional homologue of DNA-damage binding protein 1 (DDB1). DDB1 also interacts with Cul4 to function in nucleotide excision repair (Angers *et al.* 2006; Li *et al.* 2006; Scrima *et al.* 2008), and binds to a family of WD40-repeats containing proteins that are thought to act as receptors for substrates of the DDB1-CUL4 E3 machinery (Angers *et al.* 2006). Dos1 is a WD40 protein containing conserved signatures for DDB1-interacting proteins and may serve a similar function with Rik1-Cul4 as part of CLRC. In filamentous fungus *Neurospora crassa*, a H3K9 methyltransferase DIM-5 has been shown to form a multiprotein complex with DDB1-CUL4. Moreover, this interaction is also mediated by proteins related to Dos1 and Dos2 (Lewis *et al.* 2010). It is thus conceivable that CLRC may represent a scenario in which modification of Clr4 by Rik1-Cul4-Dos1 promotes its recruitment to DNA or H3K9me activity at target regions.

Rik1-Dos1 can directly interact with a JmjC-domain H3K4 demethylase, Lid2, that is thought to bring CLRC to the pericentromeric repeats. Hypomethylation of H3K4 by Lid2 is a required step that precedes H3K9me by Clr4 for heterochromatin formation (Li *et al.* 2008). The subsequent recruitment of RITS/RNAi to these regions can then be explained by the association of Chp1 with H3K9me. Although RNAi is not absolutely required to recruit CLRC to the pericentromeric repeats or for CLRC-mediated heterochromatin formation (Jia *et al.* 2004; Kagansky *et al.* 2009), RNAi is required for spreading of CLRC activity across heterochromatin and transgenes within these regions, suggesting an additional interaction exists for RNAi recruitment of CLRC within the centromere (Volpe *et al.* 2002; Bayne *et al.* 2010).

## A complex interaction

**Fig. 3:**
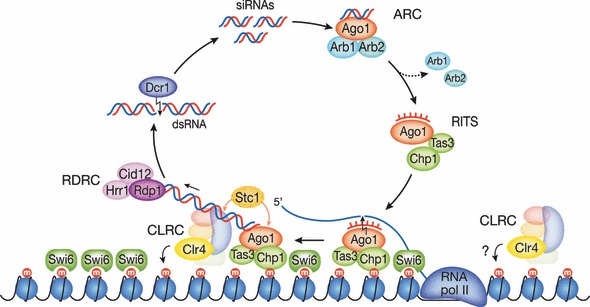
Schematic model of the self-enforcing loop for RNAi-mediated epigenetic silencing in *Schizosaccharomyces pombe*. Heterochromatin is shown as DNA (black line) wrapped around nucleosomes (blue circles) modified by histone H3 lysine 9 methylation (H3K9me; red circles “m”). Nascent transcripts generated by RNA polymerase II are targeted by an activated RNAi-induced transcriptional silencing (RITS) complex that also interacts with H3K9me through the Chp1 subunit. RDRC is then recruited for synthesis of dsRNA from the transcripts, which is cleaved into duplex siRNAs by Dcr1 and bound by Argonaute siRNA chaperone (ARC). These siRNAs are then processed into single-stranded siRNAs and loaded back into RITS completing the loop. Catalytically active RITS recruits the CLR4 complex (CLRC) for spreading of epigenetic silencing into adjacent regions through interaction with Stc1, although CLRC can also associate with these regions independent of RNAi and recruit inactive RITS.

The relationship between CLRC and RITS/RNAi exemplifies the presence of a self-enforcing loop for maintenance of heterochromatin and epigenetic silencing at *S. pombe* centromeres. CLRC is required upstream of RNAi, however, slicing activity of Ago1 at transcripts is also required to recruit CLRC and H3K9me into non-repeat sequences. The maintenance of epigenetic silencing at heterochromatin therefore requires cross-talk between complexes with specific functions. These can be direct interactions between complex subunits, or involve additional proteins that play important regulatory roles. A schematic model of the self-enforcing loop based on current knowledge of the relevant complexes is shown in Figure 3.

### Interactions within the self-enforcing loop

Of the four main complexes described above, RITS and CLRC are thought to be tightly associated with chromatin, whereas RDRC appears to have a more peripheral association involving nascent transcripts and ARC has a wider distribution throughout the cell. In a wild-type cell, one strand of the pericentromeric repeats is transcribed and turned over when all complexes are functioning normally. These nascent transcripts serve as a basal point for the self-enforcing loop and as substrate for siRNAs that participate in silencing. Transcription of pericentromeric repeats is carried out by RNA polymerase II (RNAPII) (Djupedal *et al.* 2005; Kato *et al.* 2005), although the contribution of RNAPII in epigenetic silencing appears to be more than just providing the transcript substrate. A mutation in the largest subunit of RNAPII resulted in loss of siRNAs corresponding to the repeats and disrupted heterochromatin at transgenes within the pericentromeric region. However, this mutation did not affect RNAPII localization to the pericentromeric region and transcripts from the repeats also accumulated similar to that observed in RNAi mutants (Kato *et al.* 2005). This suggested that loss of epigenetic silencing was not due to absence of transcript template but rather that RNAPII interacts with RITS to enable coupling of transcription with RNAi-mediated epigenetic silencing.

The RITS complex interacts with nascent transcripts from the pericentromeric regions, and the activity of RITS in processing these transcripts is thought to be guided by complementary single-stranded siRNAs loaded into Ago1 (Buker *et al.* 2007). RITS also directly interacts with Clr4-methylated H3K9me through the Chp1 subunit (Noma *et al.* 2004; Sadaie *et al.* 2004; Verdel *et al.* 2004). It is expected that competition exists between Swi6 (HP1) and Chp1 (RITS) for binding the H3K9me target site. Although the number of Chp1 proteins is lower than that of Swi6, the affinity of Chp1 for H3K9me is approximately 10-fold greater than that of Swi6 (Schalch *et al.* 2009) and thus RITS can likely access H3K9me when necessary due to dynamic association of Swi6.

In the next step of the loop, RITS interacts with both RDRC and CLRC through its Ago1 subunit (Motamedi *et al.* 2004; Bayne *et al.* 2010). When RITS is activated, the interaction with RDRC promotes synthesis of dsRNA based on the target transcript, whereas the interaction with CLRC promotes further H3K9me of the surrounding chromatin. Both of these interactions are dependent on Ago1 catalytic activity and siRNAs, raising the question of how specific interactions with RNA targets and two distinct protein complexes can be achieved by a single Ago1 protein (Motamedi *et al.* 2004). It can be expected that additional factors are supporting the complex interactions. Evidence for this was recently provided by the discovery of the LIM domain-containing protein, Stc1, that can bind both CLRC and Ago1 but not RDRC (Bayne *et al.* 2010). The interaction between CLRC and Ago1 was dependent on the presence of Stc1, suggesting it functions as a mediator for RITS recruitment of CLRC. Deletion of Stc1 also caused a strong reduction in centromeric siRNA accumulation and disrupted the association between RITS and RDRC, consistent with it having a function to also support RITS activity at the repeats.

The dsRNA products provided by RDRC activity then proceed through the loop to function as substrates for Dcr1 and are processed into siRNAs. The mechanism by which dsRNA is released from RDRC and interacts with Dcr1 remains unclear, although it may have parallels to that for transcription as suggested by the presence of polyA polymerase subunit in RDRC and contribution of splicing components (Motamedi *et al.* 2004; Bayne *et al.* 2008; Chinen *et al.* 2010). Dcr1 has also been found to associate with RDRC (Colmenares *et al.* 2007), suggesting that the synthesis and transfer of dsRNA may be partially coupled. However, similar to that for interactions between RITS, CLRC and RDRC, it can be assumed that additional proteins yet to be identified are also mediating the interaction between RDRC and Dcr1. The duplex siRNAs produced by Dcr1 are then fed into ARC, which mediates processing of the siRNA into a single-strand of the correct length for loading into RITS (Buker *et al.* 2007). At present it is not known if the same Ago1 molecule shuttles between ARC and RITS permitting siRNA maturation by Ago1 slicing activity as the complex composition changes, or whether a mechanism is in place to transfer siRNAs between the two separate Ago1-containing complexes and remove the siRNA passenger strand. Whichever the mechanism, this final step results in a chromatin-associated active RITS complex and a return to the beginning of the self-enforcing loop.

It is important to emphasize that the complexes described here should not be considered as fixed, stable complexes. One example of this is the ability of CLRC subunits Rik1 and Dos1 to associate and interact with chromatin even in the absence of Clr4 (Bayne *et al.* 2010). Indeed, it has been proposed that this complex may function in a stepwise fashion with the Rik1-Dos1 subunits first interacting with a H3K4 demethylase and then bringing Clr4 to the chromatin for H3K9me (Li *et al.* 2008). Both the complexes themselves and the supporting interactions between them are thought to be highly dynamic with constant exchange of subunit components and in various states of assembly.

### Interactions activating the self-enforcing loop

The concept of the self-enforcing loop is based on the interdependent relationship between Clr4 activity and the RNAi pathway. Clr4 is required for the accumulation of high siRNA levels and the mutual interaction between RNAi complexes (Motamedi *et al.* 2004; Noma *et al.* 2004; Hong *et al.* 2005; Buhler *et al.* 2006; Halic & Moazed 2010). On the other hand, the RNAi pathway and siRNA products are required for H3K9me at centromeric repeats and inserted marker loci (Volpe *et al.* 2002). However, the recruitment or targeting of complex components to chromatin does not exclusively depend on this self-enforcing loop. CLRC can associate with chromatin independent of the RITS complex or RNAi (Jia *et al.* 2004). In addition, RITS can still associate with heterochromatin independent of Ago1 catalytic activity (Irvine *et al.* 2006). More recently, it was reported that Dcr1 and low levels of the RDRC component Rdp1 could also bind to centromeric repeats independent of Clr4 activity (Woolcock *et al.* 2011).

The process for *de novo* formation of heterochromatin that leads into the self-enforcing loop is one aspect that requires further investigation. Low levels of H3K9me persist at the centromeric repeat regions in RNAi-deficient cells (Sadaie *et al.* 2004) and play an essential role in the initial step of heterochromatin formation (Partridge *et al.* 2007). These results suggest that an RNAi/siRNA-independent mechanism provides an initial H3K9me mark at the centromeric repeat repeats and this drives the self-enforcing loop. However, this idea was challenged by the discovery of Dicer- and Rdp1-independent small RNAs, called primal RNAs (priRNAs), that associated with Ago1 in *S. pombe* (Halic & Moazed 2010). Based on a comparison of H3K9me levels in mutants lacking Dcr1, Ago1 and Clr4, it was proposed that these priRNAs play a role promoting H3K9me, and that RNAi-dependent factors initiate *de novo* heterochromatin assembly. However, another report argues against an upstream role for siRNAs. Using a genetic approach to transiently deplete CLRC or RNAi components, it was shown that Ago1 was not required to initiate *de novo* H3K9me at centromeres by Clr4 (Shanker *et al.* 2010). Although it is seemingly difficult to assemble these contradictory results, a plausible idea would be that different heterochromatic regions use distinct pathway to establish primal marks that drive the self-enforcing loop. This is supported by the finding that Clr4 has activity against substrates other than H3K9 and that this alternative activity is able to promote siRNA production from one of the two types of endogenous pericentromeric repeats (Gerace *et al.* 2010).

### Interactions with the cell cycle and DNA replication

Similar to the fact that epigenetic silencing complexes are thought to be highly dynamic, consideration also needs to be made of the dynamic nature of chromatin and how RNAi-mediated epigenetic silencing interacts with other molecular processes that occur along the chromosome. One chromatin remodeling process expected to conflict with the self-enforcing loop is that of DNA replication. Centromeric transcripts and siRNAs accumulate predominantly during S-phase, and it is at this point that H3K9me needs to be transferred or spread along the newly synthesized DNA (Chen *et al.* 2008; Kloc *et al.* 2008). This suggests that both RNAi-mediated silencing and DNA replication machineries are required to function within the repeats at the same time. Surprisingly, a direct interaction between DNA replication factors and the epigenetic silencing factor Rik1 has recently been found (Li *et al.* 2011; Zaratiegui *et al.* 2011).

Rik1 and Dos2 have been identified as part of a second complex separate from CLRC, which contains the transcriptional activator Mms19 and DNA polymerase subunit Cdc20 (Li *et al.* 2011). This complex, referred to as the Rik1-Dos2 complex (Fig. 2), promotes the transcription of pericentromeric repeats during S-phase. The presence of Rik1 in this complex is presumed to result in subsequent recruitment of CLRC for spreading of H3K9me across both chromatids of the newly replicated DNA. However, spreading of H3K9me via the self-enforcing loop is dependent on transcription of the repeats by RNAPII and it was unclear how this could be achieved since transcription and replication at the same location would conflict and cause the replication fork to stall. It has now been shown that this conflict is overcome by the RNAi-dependent processing of nascent transcripts that, while recruiting heterochromatin formation through the self-enforcing loop, promotes degradation of the transcripts and releases RNAPII (Zaratiegui *et al.* 2011). Removal of RNAPII enables the replication fork to continue and the chromatin remodeling machinery to spread H3K9me into the replication fork. Without this RNAi processing of the transcripts, the stalled replication forks are interpreted as sites of DNA damage and enter into a homologous recombination-mediated repair pathway that causes both the Rik1 complexes and epigenetic silencing to be lost.

## Perspectives

Our understanding of the major players and effector complexes for RNAi-mediated epigenetic silencing and heterochromatin assembly has advanced considerably over the last 10 years. However, as new components and functions are discovered, it becomes apparent that that there are still many questions that remain unanswered. Examples of this are Ago1 and Rik1, which are two core proteins that are located in multiple complexes. The identification of ARC provided a concept for siRNA loading of Ago1 in RITS, but it remains to be determined how siRNA transfers between the two complexes, whether the same Ago1 molecule shuttles between the two complexes, and the extent that separate functions for Ago1 are controlled by its other subunits. Rik1 was one of the first genes to be implicated in heterochromatin formation and is now known to form two distinct complexes. These complexes explain the critical role for Rik1 and how multiple processes of transcription, chromatin modification and replication are intertwined. However, the nature of Rik1 contribution to these complexes still requires clarification and the dynamics behind subunit interactions remain vague. The HP1 protein Swi6 gained prominence as a signal for completion of typical heterochromatin formation irrespective of RNAi involvement and its role was thought to be clear. However, Swi6 has now been shown to participate in the initiation of replication within pericentromeric repeats (Hayashi *et al.* 2009) and to be required for efficient RNAi processing of centromeric transcripts (Motamedi *et al.* 2004, 2008; Buhler *et al.* 2006; Halic & Moazed 2010). Much research is still required before a complete picture of heterochromatin assembly can be drawn.

A central question for future research to understand heterochromatin formation is what exactly defines the region to be targeted for epigenetic silencing. It has been eloquently demonstrated that tethering of Clr4 is sufficient to trigger formation of heterochromatin, and importantly, that the RNAi pathway is dispensable for assembling of this silent heterochromatin into a functional centromere (Kagansky *et al.* 2009; Bayne *et al.* 2010). This appears to suggest that the primary purpose of RNAi processing in heterochromatin is to recruit Clr4 methyltransferase, which then raises the question of what type of chromatin signature is destined to be targeted by Clr4. Several lines of evidence suggest that genes with convergent transcripts are marked by H3K9me and Swi6 (Gullerova & Proudfoot 2008), whereas another study showed that Clr4 also plays a critical role in degradation of read-through transcripts from non-heterochromatic coding sequences (Zofall *et al.* 2009; Zhang *et al.* 2011). While it is difficult to determine whether these typically euchromatic regions have formed heterochromatin, these observations imply that unfavorable transcriptional states are targeted by Clr4 activity. The simplest conclusion from these observations is that double stranded RNA formed by read-through transcripts are processed and targeted by the RNAi pathway to recruit Clr4. Considering that Rik1 shows structural similarity to DDB1 that functions in DNA repair and to CPSF-160 that functions in pre-mRNA 3′ processing (Mandel *et al.* 2008), the Rik1-containing CLRC complex may also target chromatin by recognizing structural DNA or RNA signatures representative of unfavorable transcription.
